# First detection of *Wolbachia*-infected *Culicoides* (Diptera: Ceratopogonidae) in Europe: *Wolbachia* and *Cardinium* infection across *Culicoides* communities revealed in Spain

**DOI:** 10.1186/s13071-017-2486-9

**Published:** 2017-11-23

**Authors:** Nonito Pagès, Francesc Muñoz-Muñoz, Marta Verdún, Núria Pujol, Sandra Talavera

**Affiliations:** 1 Institut de Recerca i Tecnologia Agroalimentàries (IRTA), Centre de Recerca en Sanitat Animal (CReSA), Campus de la Universitat Autònoma de Barcelona, (Cerdanyola del Vallès), 08193 Bellaterra, Barcelona Spain; 2 Present address: CIRAD, UMR ASTRE, F-97170 Petit-Bourg, Guadeloupe France; 3 Present address: ASTRE, Univ Montpellier, CIRAD, INRA, Montpellier, France; 4 Departament de Biologia Animal, de Biologia Vegetal i d’Ecologia, Universitat Autònoma de Barcelona, 08193 Bellaterra, Barcelona Spain

**Keywords:** Endosymbionts, *Wolbachia*, *Cardinium*, *Culicoides*, Vector control, Disease

## Abstract

**Background:**

Biting midges of the genus *Culicoides* (Diptera: Ceratopogonidae) transmit pathogens that cause important diseases. No effective technique has been found to properly control either *Culicoides* spp. abundance or their likelihood to transmit pathogens. Endosymbionts, particularly *Wolbachia*, represent powerful alternatives to control arthropods of health interest. In arthropods, *Wolbachia* can reduce vector fitness and vector’s pathogen transmission capacity, thus being a potential target for population reduction and replacement strategies.

**Results:**

The presence of *Wolbachia* and *Cardinium* endosymbionts was screened in Spanish *Culicoides* spp. populations at livestock premises and natural habitats. The first detection of *Wolbachia*-infected *Culicoides* spp. in Europe is reported. The putative Palaearctic vectors for bluetongue and Schmallenberg diseases, *C. imicola*, *C. obsoletus* (*s*.*s*.) and *C. pulicaris* (*s*.*l*.), were infected with *Wolbachia*. Four genetic clusters of closely-related *Wolbachia* strains from A and B supergroups were detected infecting *Culicoides. Cardinium* strain of the C-group was detected in *C. obsoletus* (*s*.*l*.). Both endosymbionts, *Wolbachia* and *Cardinium*, were detected in *Culicoides* species of minor epidemiological relevance as well. Higher prevalence of *Wolbachia* infection was detected in natural habitats, while livestock premises lead to higher prevalence of *Cardinium*. Significant differences in the prevalence of *Wolbachia*, but not *Cardinium*, were also detected between some *Culicoides* species and between locations.

**Conclusions:**

The presence of *Wolbachia* and *Cardinium* endosymbionts in *Culicoides* is expected to trigger new research towards the control of *Culicoides*-transmitted diseases. The results of the present study could have an impact beyond the *Culicoides* arena because successful *Wolbachia* transfection is possible even across genus and species barriers.

**Electronic supplementary material:**

The online version of this article (10.1186/s13071-017-2486-9) contains supplementary material, which is available to authorized users.

## Background

Biting midges of the genus *Culicoides* (Diptera: Ceratopogonidae) are well known worldwide for transmitting pathogens that cause important diseases. In Europe, *Culicoides* became a major concern for spreading the largest Bluetongue (BT) epizootics ever recorded [1]. Since 1998, BT was reintroduced, and recurrent outbreaks are still ongoing. Through BT disease, *Culicoides* provoked major economic losses with important disruption of international animal trade [2, 3]. In addition, *Culicoides* are involved in the spread of other important arboviral diseases such as African horse sickness [4], Epizootic haemorrhagic disease [4], and the recently emerged Schmallenberg disease [5, 6]. *Culicoides* are also vectors for pathogens of different aetiology, such as filarial worms and protozoa [4]. To date, no effective vector control technique or approach to limit the likelihood of pathogen transmission has been found for *Culicoides* [7, 8].

Endosymbiotic bacteria are naturally found in insects [9]. In recent years, the growing number of field screenings in several arthropod phyla led to an increase in the reported endosymbiont prevalence in arthropods [10]. A meta-analysis performed by Hilgenboecker et al. [11] suggested up to 66% of all insect species were infected with *Wolbachia* (class Alphaproteobacteria, order Rickettsiales). *Wolbachia* is an obligate intracellular endosymbiotic bacteria present in a wide range of arthropods and filarial nematodes worldwide. *Wolbachia* and other bacterial endosymbionts are well known as master manipulators of arthropod host reproduction [12]. Such endosymbionts can manipulate host reproduction by inducing cytoplasmic incompatibility of host gametes, feminization of genetic males, parthenogenesis and male-killing [13, 12]. The endosymbiont *Cardinium* (*Cytophaga*-*Flavobacterium*-*Bacteroides*) is another well-characterised endosymbiont of arthropods. *Cardinium* has a lower infection prevalence and is restricted, apparently, to fewer taxonomic groups [14].

Research on arthropod endosymbionts is strongly consolidated for mosquitoes [15]. This challenge has been addressed for other important arthropod vectors such as tsetse flies [16]. Despite its potential interest, the prevalence of endosymbionts across *Culicoides* communities and ecosystems has been scarcely studied. However, recent studies have endorsed the presence of endosymbionts in *Culicoides*. First attempts were performed by Nakamura et al. [17] in Asia. The authors reported, for the first time, *Culicoides* midges infected with *Wolbachia* and “*Candidatus* Cardinium hertigii” (Bacteroidetes). The latter led to the description of a new *Cardinium* group (group C). Soon after, Morag et al. [18] described *Cardinium*-infected *Culicoides* in Israel. More recently, Lewis et al. [19] detected the same endosymbiont in *Culicoides* in the United Kingdom. *Cardinium* DNA sequences of the strains reported in the three previous studies are highly conserved. More recently, *Wolbachia* and *Cardinium* were detected in *Culicoides* species from the Australasian region and Africa [20]. A recent study confirmed the absence of *Wolbachia* in feminized males of the species *C. circumscriptus* collected at ten populations from Spain [21]. To date, endosymbionts of the genus *Wolbachia* remain undetected in European *Culicoides* communities.

Use of endosymbionts, *Wolbachia* particularly, is a promising approach for controlling the dynamics of some arthropod-transmitted pathogens under certain scenarios. They have the potential to modulate major parameters of arthropod vectorial capacity. Thus, infection with certain *Wolbachia* strains can lead to a decrease in arthropod survival as shown in *Aedes aegypti* [22]. Moreover, *Wolbachia* can influence arthropod’s vector competence of important pathogens. For example, *Wolbachia*-infected *Anopheles stephensi* became refractory to infection with *Plasmodium falciparum* [23]. *Wolbachia* has been studied as well for its potential to introduce transgenes into arthropod natural populations [24], and more interestingly within vectors of arthropod borne diseases [25]. *Wolbachia* and (indirectly) the mitochondrial genome of its host can rapidly invade and establish through uninfected populations by manipulating its host-reproduction with the mechanisms mentioned above [13].

The present study examined whether *Wolbachia* and *Cardinium* bacterial endosymbionts naturally infected *Culicoides* species communities across Spanish ecosystems. Once confirmed, *Wolbachia* and *Cardinium* strains were genotyped. Then, the effect of *Culicoides* species, geographical origin and habitat type on endosymbiont prevalence was assessed.

## Methods

### Sampling

**Fig. 1:**
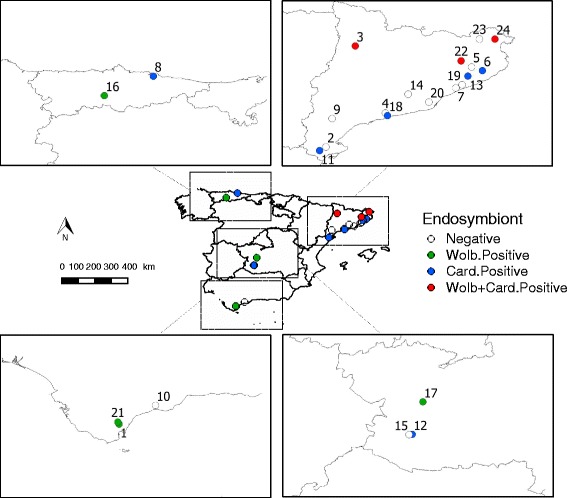
Sampling sites in Spain where *Culicoides* spp. were tested for the presence of endosymbionts. Sampling sites: 1, Almoraima; 2, Amposta; 3, Aramunt; 4, Bonastre; 5, Brunyola; 6, Caldes de Malavella; 7, Canyamars; 8, Colunga; 9, Garcia; 10, Juanar; 11, La Galera; 12, La Morera; 13, Massanas; 14, Piera; 15, Pozo Estanco; 16, Proaza; 17, Quintos de Mora; 18, Roda de Barà; 19, Sant Iscle de Vallalta; 20, Sant Just Desvern; 21, Santa Clara; 22, Susqueda; 23, Terrades; 24, Vilanova de la Muga

Biting midges were captured at four Spanish NUTS2 (*Nomenclature des unités territoriales statistiques*) administrative units: Catalonia, Asturias, Castilla La Mancha and Andalucía. Twenty-four sites were sampled across the Iberian Peninsula (Fig. 1, Table 1). At each NUTS2, sampling sites included two habitat types, livestock premises and natural habitats (Table 1). Natural habitats consisted of forestry areas usually located more than 1 km far from the closest livestock farm. *Culicoides* collections were made between the years 2009–2012. Biting midges were trapped using Center for Disease Control (CDC) Miniature ultraviolet (UV)-light traps (model 912, John W. Hock Company, Gainesville, USA) in soapy water during the night. *Culicoides* were recovered the day after and transferred to 70% ethanol. *Culicoides* were identified under a stereomicroscope (Nikon model SMZ) according to their pattern of wing pigmentation [26] and stored frozen (-20 °C) for further downstream processing. A map including *Culicoides* collection sites and endosymbionts spatial distribution was prepared with Quantum GIS software [27] using the coordinate reference System (CRS) EPSG:4326, WGS 84.

**Table 1:** *Culicoides* spp. sampling sites used for endosymbiont screening

Code	Location	Habitat	NUTS2	Coordinates	Altitude (m)
1	Almoraima	Natural	Andalucía	36.28819°N, -5.43164°E	29
10	Juanar	Natural	Andalucía	36.57012°N, -4.89140°E	870
21	Santa Clara^a^	Livestock premise	Andalucía	36.32058°N, -5.45014°E	157
8	Colunga	Natural	Asturias	43.48888°N, -5.34220°E	203
16	Proaza	Natural	Asturias	43.20327°N, -6.05850°E	349
12	La Morera	Natural	Castilla Mancha	38.90910°N, -4.27157°E	718
15	Pozo Estanco^a^	Livestock premise	Castilla Mancha	38.90585°N, -4.31681°E	677
17	Quintos de Mora	Natural	Castilla Mancha	39.39248°N, -4.11572°E	707
2	Amposta	Livestock premise	Catalunya	40.72827°N, 0.55853°E	28
3	Aramunt	Livestock premise	Catalunya	42.20605°N, 0.98767°E	559
4	Bonastre	Livestock premise	Catalunya	41.22280°N, 1.42745°E	210
5	Brunyola	Livestock premise	Catalunya	41.90096°N, 2.69078°E	194
6	Caldes de Malavella	Livestock premise	Catalunya	41.84400°N, 2.84987°E	120
7	Canyamars	Livestock premise	Catalunya	41.59342°N, 2.46252°E	240
9	Garcia	Livestock premise	Catalunya	41.14453°N, 0.64853°E	65
11	La Galera	Livestock premise	Catalunya	40.67528°N, 0.46552°E	102
13	Massanas	Livestock premise	Catalunya	41.76520°N, 2.63887°E	100
14	Piera	Livestock premise	Catalunya	41.50088°N, 1.76163°E	324
18	Roda de Barà^a^	Livestock premise	Catalunya	41.18704°N, 1.46041°E	59
19	Sant Iscle de Vallalta	Livestock premise	Catalunya	41.63948°N, 2.55123°E	227
20	Sant Just Desvern^a^	Livestock premise	Catalunya	41.38534°N, 2.06594°E	65
22	Susqueda	Livestock premise	Catalunya	41.98677°N, 2.53857°E	349
23	Terrades	Natural	Catalunya	42.30769°N, 2.80464°E	345
24	Vilanova de la Muga	Livestock premise	Catalunya	42.30568°N, 3.03397°E	19

^a^Coordinates and altitude inferred respectively from Google Earth (©2013 Google Inc.) and GPSvisualizer (www.gpsvisualizer.com)

*Abbreviation*: NUTS2, Classification of Territorial Units for Statistics

### DNA extraction, PCR and sequencing

Crude homogenates of individual female midges were prepared in 200 μl phosphate buffered saline (PBS), using glass beads in a Fastprep® (MP Biomedicals, Solon, USA) at a speed of 5 m/s. A fraction of 10 homogenates (20 μl each), of the same species and collection site, were pooled for DNA extraction. DNA extractions, both from pools or individuals, were performed using a commercial kit (DNeasy Blood and Tissue Kit, Qiagen, Crawley, UK) following the manufacturer’s instructions, with a final elution volume of 100 μl. Two positive controls were used. The first was obtained from a *Culicoides* infected with *Wolbachia*. The second was obtained from a *Culicoides* infected with *Cardinium*. A non-infected *Culicoides* was used as negative control.

Presence of endosymbiotic bacteria of the genus *Wolbachia* and *Cardinium* was assessed using specific single polymerase chain reaction (PCR). Briefly, the presence of *Wolbachia* was tested by amplification of a fragment of the *wsp* (*Wolbachia* surface protein) gene delimited by primers wsp81F and wsp691R [28]. The presence of “*Candidatus* Cardinium heretgii” was tested by amplification of a fragment of the 16S rDNA delimited by primers CLO-f1 and CLO-r1 [26]. The first screening was performed in pools of 10 *Culicoides*. When a pool was confirmed positive, the initial crude homogenates, corresponding to individual *Culicoides* in the positive pooled sample were used for individual DNA extraction and endosymbiont PCR detection. Positive individuals from the Obsoletus group were identified to species using a cytochrome oxidase subunit I (*cox*1) gene specific PCR test [29, 30]. PCR reactions were carried out using the GeneAmp PCR System 9700 (Applied Biosystems, Foster City, CA). Negative controls were included in every PCR. PCR products were confirmed by ethidium bromide staining after electrophoresis on 2% (*w*/*v*) agarose 1× TAE gel run at 10 V/cm for 1 h.

When considered necessary, positive samples were sequenced. PCR products were purified, and DNA purified products were sequenced on both strands using Big Dye Terminator version 3.1 cycle sequencing kit (Applied Biosystems) and analysed on an ABI PRISM 3730 Automated sequencer (Applied Biosystems).

### Phylogenetic analysis

DNA sequences were edited using Bioedit sequence alignment editor software (version 5.0.9. for Windows [31]) and aligned with ClustalW Multiple alignment option without manual optimization. Phylogenetic and molecular evolutionary analyses were conducted using MEGA6 software [32]. Phylogenetic analysis was inferred using Maximum Likelihood (ML) method incorporating best-fit models of sequence evolution determined using the Akaike information criterion with a resampling nodal support of 1000 bootstrap replicates. Best-fit models were Tamura 3-parameter (T92 + G) for *Wolbachia* dataset, and Kimura 2-parameter Gamma corrected (K2 + G) for *Cardinium*. Sequences published at the National Center for Biotechnology Information (NCBI) were used to better determine the evolutionary relationship of *Wolbachia* and *Cardinium* isolates (Additional file 1: Table S1).

### Statistical analyses

The prevalence of *Wolbachia* and *Cardinium* infection was calculated for *Culicoides* species, site, geographical region and habitat. Differences in infection prevalence were tested using the two tailed Fisher’s exact test [18]. Differences among species were tested separately in sites where more than one *Culicoides* species was captured, and at least in one of the species endosymbiont infection was detected. Differences among sites of the same administrative unit and habitat type were tested separately in species of *Culicoides* that were trapped in more than one site and at least in one of these sites positive infections were detected. Differences between habitats were tested within geographical regions both separating by *Culicoides* species and grouping all species together, and considering the whole sample together as well. All these analyses were performed separately for the prevalence of *Wolbachia* and *Cardinium* infections.

## Results

PCR screening for *Wolbachia* and *Cardinium* allowed the detection of endosymbionts in several *Culicoides* populations (Fig. 1). The most epidemiologically relevant *Culicoides* species in Europe were infected with *Wolbachia* and *Cardinium* (Table 2). No double infection was detected in the 1050 *Culicoides* analysed. However, *Wolbachia* and *Cardinium* were found in sympatry in three out of the 24 sampled sites (Fig. 1).

**Table 2:** Endosymbiont PCR screening and DNA sequencing of *Culicoides* collected at natural habitats and livestock premises in Spain. Tests results are segregated horizontally by group of *Culicoides* spp. and NUTS2 regions

Group	NUTS2	*Wolbachia*				*Cardinium*				Year collection			
		Livestock premise		Natural habitat		Livestock premise		Natural habitat					
		W+	W-	W+	W-	C+	C-	C+	C-	2009	2010	2011	2012
IMI		1	115	5	135	0	108	0	140				
	Andalucía	1 (1_Seq)	47	5 (4_Seq)	95	0	48	0	100	+		+	
	Castilla Mancha	0	50	0	40	0	50	0	40	+	+		
	Catalunya	0	18	–	–	0	10	–	–	+			
OBS		0	162	2	301	1	151	1	300				
	Andalucía	0	50	0	80	0	50	0	80		+	+	
	Asturias	0	10	2 (2_Seq)	101	0	10	1 (1_Seq)	100	+	+		
	Castilla Mancha	–	–	0	70	–	–	0	70		+		
	Catalunya	0	102	0	50	1 (1_Seq)	91	0	50	+		+	+
PUL		0	5	56	248	0	3	0	300				
	Andalucía	–	–	47 (8_Seq)	54	–	–	0	100	+			
	Asturias	–	–	2 (1_Seq)	98	–	–	0	100	+	+		
	Castilla Mancha	–	–	7 (5_Seq)	44	–	–	0	50		+	+	
	Catalunya	0	5	0	52	0	3	0	50	+		+	
Other		3	39	0	8	9	31	1	5				
	Andalucía	–	–	0	2	–	–	0	2	+			
	Asturias	–	–	0	3	–	–	0	2	+	+		
	Castilla Mancha	–	–	0	3	–	–	1	1	+	+		
	Catalunya	3 (2_Seq)	39	–	–	9 (6_Seq)	31	–	–	+	+		
Total		4	321	63	692	10	293	2	745				

*Abbreviations*: *W+* no. of *Wolbachia*-positive (PCR) tests; *W-* no. of *Wolbachia*-negative (PCR) tests; *C+* no. of *Cardinium-*positive (PCR) tests; *C-* no. of *Cardinium*-negative (PCR) tests. The number of *Culicoides* for which endosymbiont DNA sequences were obtained are shown in parentheses. Definition of species groups: IMI, *C. imicola*; OBS, *C. obsoletus* (*s.l*.); PUL, *C. pulicaris* (*s.l*.); Other; other *Culicoides* spp

### *Wolbachia* screening

*Wolbachia* infection was present in some of the putative vectors of bluetongue (BTV) and Schmallenberg (SBV) viruses in Europe: *C. imicola*, *C. obsoletus* (*s*.*l*.) and *C. pulicaris* (*s*.*l*.) *Wolbachia* infection was also detected in *C. vexans*, *C. kibunensis* and *C. heteroclitus*.

The prevalence of W*olbachia* infection detected at metapopulation scale was low for the species with a representative geographical screening at the population level: *C. imicola*, *C. obsoletus* (*s*.*l*.) and *C. pulicaris* (*s*.*l*.). In *C. imicola*, 6 out of 256 individuals were positive with a prevalence ratio (PR) of 0.023. In *C. obsoletus* (*s*.*l*.), 2 of 466 individuals tested were positive (PR = 0.004). Conversely, infection prevalence in *C. pulicaris* (*s*.*l*.) was higher, with 56 positive individuals out of the 309 tested (PR = 0.18). These results were supported by statistical tests, which indicated that prevalence of infection differed between some *Culicoides* species. The prevalence of *Wolbachia* in *C. pulicaris* (*s*.*l*.) was significantly higher than in *C. imicola* and *C. obsoletus* in two out of the three sites tested (Almoraima: *P* < 0.001 in both specific comparisons; Quintos de Mora: *P* < 0.05 in both specific comparisons). However, the prevalence of *Wolbachia* did not differ between *C. obsoletus* and *C. imicola* in any of the three sites where both species were detected in sympatry.

Our screening indicated that *Wolbachia* infection had a heterogeneous spatial distribution (Fig. 1, Table 2, Additional file 2: Table S2). The prevalence of *Wolbachia* infection in particular *Culicoides* species even differed among sites of the same geographical region. Two out of three populations of *C. imicola* from Andalucía, Almoraima and Santa Clara, were infected with *Wolbachia*. Although the prevalence of *Wolbachia* infection did not significantly differ among the three populations from Andalucía, differences between Almoraima (PR = 0.10) and Juanar (PR = 0.00) were almost significant (*P* = 0.058). None of the *C. imicola* populations screened in Castilla La Mancha, or Catalonia tested positive. *Culicoides imicola* was not collected in Asturias. *Wolbachia* infection in *C. obsoletus* (*s*.*l*.) was evident in one out of three populations of Asturias (Proaza). However, infection prevalence did not differ significantly among them. The two *Culicoides* females infected with *Wolbachia* in Proaza were genetically identified as *C. obsoletus* (*s*.*s*.). None of the *C. obsoletus* (*s*.*l*.) populations from Andalucía, Castilla la Mancha or Catalonia, tested positive. Three populations of *C. pulicaris* (*s*.*l*.) from Andalucía (Almoraima), Castilla La Mancha (Quintos de Mora), and Asturias (Proaza) were infected with *Wolbachia*. The population of *C. pulicaris* (*s*.*l*.) from Almoraima showed the highest infection prevalence detected in the study, with more than 90% of specimens positive for *Wolbachia*. The prevalence of *Wolbachia* infection detected in Almoraima was significantly higher than in the remaining populations of *C. pulicaris* (*s*.*l*.), including another population from Andalucía, Juanar (*P* < 0.001), in which *Wolbachia* was not detected.

An effect of habitat type was also detected, with a higher prevalence of *Wolbachia* infection in natural habitats than in livestock premises. However, the effect of habitat could not be detected in individual species. When grouping all the *Culicoides* species the prevalence of *Wolbachia* infection was statistically higher in natural habitats compared to that found in livestock premises in Andalucía (*P* < 0.001) and when all regions were considered simultaneously (*P* < 0.001).

### *Cardinium* screening

*Cardinium* infection was present in a wide range of species: *C. obsoletus* (*s*.*l*.), *C. festivipennis*, *C. flavipulicaris*, *C. haranti*, *C. maritimus*, *C. minutissimus*, *C. newsteadi*, *C. punctatus* and *C. sahariensis*.

Conversely, to what was observed for *Wolbachia* infections, the prevalence of *Cardinium* infection differed neither between *Culicoides* species nor between sites, mainly because of the scarce number of infected specimens. Prevalence of *Cardinium* was low for the *C. obsoletus* (*s*.*l*.) metapopulation analyzed (PR = 0.004; 454 females tested). The endosymbiont was found in two *C. obsoletus* (*s*.*l*.) populations. The first population, Colunga, was located in a forestry area of Asturias (PR = 0.02; 51 females tested). The second, Massanes, was present at a livestock farm in Catalonia (PR = 0.01; 70 females tested). Prevalence of *Cardinium* infection in other *Culicoides* species might be biased because there was a low sample size (Additional file 2: Table S2). As observed for *Wolbachia*, a significant effect of habitat type was detected for the prevalence of *Cardinium* infection. In this case, however, infection prevalence was higher in livestock premises than in natural habitats. Thus, when grouping all the *Culicoides* species, the prevalence of infection was statistically higher in livestock premises than in natural habitats in Catalonia (*P* < 0.01) and when all regions were considered simultaneously (*P* < 0.001).

### Phylogenetic analyses of endosymbionts

#### Wolbachia

**Fig. 2:**
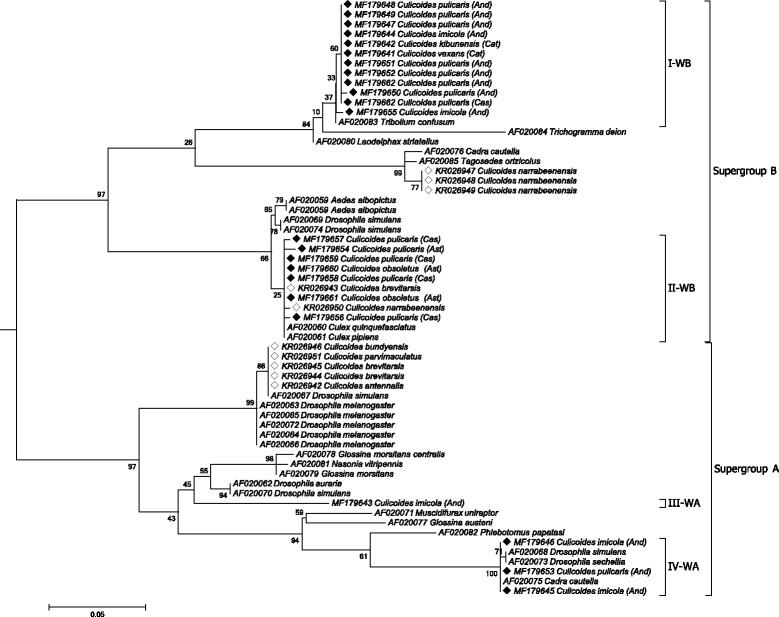
Cladogram for *Wolbachia* phylogenetic relationships inferred from *wsp* gene sequences; sequences already known from *Culicoides* are indicated with white diamonds; new *wsp* gene sequences of *Wolbachia*-infected *Culicoides* obtained in the present study are indicated with *black* diamonds. Accession numbers followed by host species are shown at terminal nodes. The tree is drawn to scale, with branch lengths measured in the number of substitutions per site. Maximum likelihood (ML) bootstrap support values over 50% are displayed on the nodes. *Abbreviations*: I-WB, clade I-*Wolbachia* supergroup B; II-WB, clade II-*Wolbachia* supergroup B; III-WA, clade III-*Wolbachia* supergroup A; IV-WA, clade IV-*Wolbachia* supergroup A; And, Andalucía; Ast, Asturias; Cas, Castilla la Mancha; Cat, Catalonia

*Wolbachia* DNA partial sequences of the *wsp* gene were obtained for 23 *Wolbachia*-infected *Culicoides* out of 67 (GenBank: MF179641–MF179663). The DNA dataset was representative of all *Culicoides* taxonomic groups that were found infected with *Wolbachia* in each geographical area (Fig. 1, Table 2). Nine haplotypes were detected among the 23 *wsp* gene sequences, with haplotype diversity (Hd) of 0.787 and nucleotide diversity (π) of 0.0916. Phylogenetic analyses placed *wsp* sequences derived from Spanish *Culicoides* within the *Wolbachia* supergroups A (*n* = 4) and B (*n* = 19). Spanish isolates were grouped into four clades, two into supergroup A and two into supergroup B (Fig. 2). *Wolbachia* supergroup A sequences were grouped in two clades, III-WA and IV-WA, and were from *Culicoides* collected in Andalucía (Table 3). Isolates of both clades infected *C. imicola* whereas a single isolate of IV-WA infected *C. pulicaris* (*s*.*l*.) as well. *Wolbachia* supergroup B sequences were grouped in two distant clades, I-WB and II-WB (Fig. 2). The first clade (I-WB) had *Wolbachia* sequences isolated from *C. imicola*, *C. kibunensis*, *C. pulicaris* (*s*.*l*.) and *C. vexans* collected at Andalucía, Catalonia and Castilla la Mancha (Table 3). Clade II-WB was representative of *C. pulicaris* (*s*.*l*.) and *C. obsoletus* (*s*.*l*.) infections (from Asturias and Castilla la Mancha) and contained sequences previously detected in Australian *Culicoides* [20].

**Table 3:** Geographical distribution of four *Wolbachia* clades isolated from *Culicoides* spp.

NUTS2	Site	*Culicoides* spp.	III-WA	IV-WA	I-WB	II-WB
Andalucía	Total		1	3	9	0
	Almoraima	*C. imicola*	1	2	1	
		*C. pulicaris* (*s.l*.)		1	7	
	Santa Clara	*C. imicola*			1	
Asturias	Total		0	0	0	3
	Proaza	*C. obsoletus* (*s.s*.)				2
		*C. pulicaris* (*s.l*.)				1
Castilla la Mancha	Total		0	0	1	4
	Quintos de Mora	*C. pulicaris* (*s.l*.)			1	4
Catalonia	Total		0	0	2	0
	Aramunt	*C. vexans*			1	
	Susqueda	*C. kibunensis*			1	
Total			1	3	12	7

*Abbreviations*: *III-WA* clade III-*Wolbachia* supergroup A; *IV-WA* clade IV-*Wolbachia* supergroup A; *I-WB* clade I-*Wolbachia* supergroup B; *II-WB* clade II-*Wolbachia* supergroup B

Sympatric infections with *Wolbachia* from clades III-WA, IV-WA and I-WB were detected at a *C. imicola* population from Almoraima (Andalucía; Table 3). At the same collection site, the *C. pulicaris* (*s*.*l*.) population was infected with *Wolbachia* from clades IV-WA and I-WB. Moreover, *C. pulicaris* (*s*.*l*.) from Quintos de Mora (Castilla la Mancha) were infected with *Wolbachia* belonging to clades I-WB and II-WB.

*Wolbachia* sequences previously detected in Australian *Culicoides* spp. [20] were segregated into two separated clades within supergroup B and a third clade into supergroup A (Fig. 2). The first clade contained three sequences of the species *C. narrabeensis* (GenBank: KR026947–KR026949) and was the phylogenetically related to clade I-WB (evolutionary distance, ED = 0.161). A sequence from *C. narrabeensis* (GenBank: KR026950) and one from *C. brevitarsis* (GenBank: KR026943) were included into clade II-WB. The last group of *Wolbachia wsp* gene sequences detected in Australian *Culicoides* were placed in a third clade into supergroup A (*C. brevitarsis*, GenBank: KR026944, KR026945; *C. antennalis*, KR026942; *C. bundyensis*, KR026946; *C. parvimaculatus*, KR026951). This clade was relatively distant from clades III-WA (ED = 0.182) and IV-WA (ED = 0.206).

Within each of the four clades, nucleotide variation was minor or absent regardless *Culicoides* species or geographical location (Fig. 2). Within clades, evolutionary distances were 0.001 (I-WB), 0.004 (II-WB) and 0 (IV-WA).

#### Cardinium

**Fig. 3:**
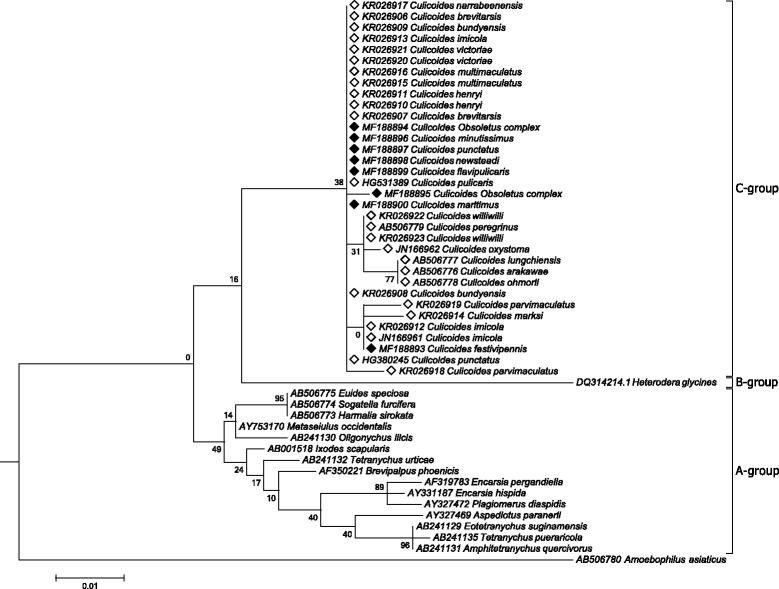
Cladogram for *Cardinium* phylogenetic relationships inferred from 16S sequences. *Cardinium* 16S rDNA sequences obtained in the present study are indicated with *black* diamonds. Accession numbers followed by host species are shown at terminal nodes. *A. asiaticus* has been used as outgroup to root the cladogram. The tree is drawn to scale, with branch lengths measured in the number of substitutions per site. Maximum likelihood (ML) bootstrap support values over 50% are displayed on the nodes

A fragment (416 bp) of the 16S ribosomal DNA gene was obtained from 8 *Cardinium-*infected *Culicoides* midges out of 12 (GenBank: MF188893–MF188900). The DNA dataset was representative of all *Culicoides* taxonomic groups that were infected with *Cardinium* in each geographical area (Fig. 1, Table 2). Nucleotide diversity among the eight *Cardinium* 16S RNA gene sequences was low (π = 0.0008), with two haplotypes differing by a singleton (Hd = 0.333). When analysed with previously published sequences of *Cardinium* strains detected in *Culicoides* (*n* = 26, Additional file 1: Table S1), the new sequence data exhibited low genetic variation (π = 0.0073, Hd = 0.504). However, the cladogram showed that all *Cardinium* sequences derived from *Culicoides* biting midges (*n* = 32) were grouped in a single clade (Fig. 3). This clade formed the recently described *Cardinium* C-group, attributed exclusively to *Culicoides* infections [17]. Additional *Cardinium* sequences from other arthropod groups and nematodes were used to infer better phylogenetic relationships among *Cardinium*. *Cardinium* B-group was derived from a strain detected in nematodes. *Cardinium* A-group was constituted by *Cardinium* strains isolated from different arthropod groups (Fig. 3).

## Discussion

To the best of our knowledge, the present study represents the first detection of *Wolbachia* infecting *Culicoides* in Europe. Endosymbionts of the genera *Wolbachia* and *Cardinium* have been detected in the putative Palaearctic vectors of BTV and SBV.

Previous studies have detected *Cardinium* endosymbionts in relevant Palaearctic *Culicoides* species, i.e. *C. imicola* [18], *C. pulicaris* and *C. punctatus* [19], usually with an intermediate prevalence. The role for *Cardinium* infection in arthropod’s fitness remains uncertain to date. In *Culicoides*, no difference in survival rate was attributed to *Cardinium* infection for *C. imicola* in laboratory conditions [33]*.* However, the present study widens the known *Cardinium* infection host range within the genus *Culicoides*. The most relevant species infected with *Cardinium* was *C. obsoletus* (*s*.*l*.). The absence of *Cardinium* infection in *C. imicola* contrasts with results obtained in Israel [17] and Africa [19]. However, *Cardinium* infection might occur in *C. imicola* non-tested populations.

*Wolbachia* was present in *Culicoides* species of major epidemiological relevance: *C. imicola*, *C. obsoletus* (*s*.*l*.) and *C. pulicaris* (*s*.*l*.). However, *Wolbachia* was detected in other *Culicoides* species of less epidemiological relevance as well. Statistical analyses showed that the prevalence of *Wolbachia* infection diverges among some species of *Culicoides*. In all cases, the species that showed the highest infection prevalence was *C. pulicaris* (*s*.*l*.), with differences in prevalence being statistically significant when compared both with *C. imicola* and *C. obsoletus* (*s*.*l*.). While the infection prevalence of these two species did not diverge when compared at the same site, differences were marginally significant in the Andalucía region (result not shown). Prevalence of *Wolbachia* infection did not only diverge among species, but also among localities. The species *C. pulicaris* (*s*.*l*.) showed the largest geographical range for *Wolbachia* infection. *Wolbachia*-infection in *C. pulicaris* (*s*.*l*.) was present in Andalucía, Asturias and Castilla La Mancha. Also, one of the populations (*C. pulicaris* from Almoraima; Additional file 2: Table S2) exhibited a high prevalence of infection, being close to fixation. Statistical analyses also indicated significant differences between habitats. In particular, prevalence of *Wolbachia* infection was higher in natural habitats than in livestock premises. However, these differences were mainly due to the high prevalence detected in *C. pulicaris* (*s*.*l*.) from one of the natural habitats (Almoraima). In contrast, the prevalence of *Cardinium* infection was higher in livestock premises than in natural habitats. These results might suggest a negative association between the two endosymbionts in the *Culicoides* communities. In fact, no double infection was detected among *Culicoides*, and although sympatric *Wolbachia* and *Cardinium* infections were detected, none of the infections affected the same *Culicoides* species. Further analyses are needed to ensure a possible effect of habitat type on the prevalence of endosymbionts and their relationship.

The large geographical range for *Wolbachia-*infected *Culicoides* detected in Spain suggests that infections may be found in other regions of Europe. A similar situation would be expected for *Cardinium* infections. Also, the recently discovered presence of *Wolbachia* or *Cardinium* low level infections in *Culicoides* [20] strongly suggests the possibility for a higher prevalence of infection than the one here reported. The presence of such low level infections in *Culicoides*, even beyond the diagnostic sensitivity of the technique we used, cannot be ruled out. Future studies should use more sensitive techniques to detect low-level infections to ascertain *Wolbachia* (and other endosymbionts) incidence and prevalence across *Culicoides* populations.

The genus *Wolbachia* is highly diverse. Most insect-infecting *Wolbachia* belong to supergroups A and B, while C and D are found in filarial nematodes, and indirectly in arthropods harbouring such nematodes [13]. Some *Wolbachia* isolates we detected represented new strains detected in *Culicoides*. *wsp* gene sequences were grouped in four clades within *Wolbachia* supergroups A and B. Based on the cladogram, the diversity of *Wolbachia* infecting *Culicoides* is expected to be complex at a global scale. The phylogenetic analysis including sequences for the few known *Wolbachia* infecting *Culicoides* revealed up to six well-defined clades. Some of the sequences were highly divergent as shown by the separation of the clades into different *Wolbachia* supergroups (A or B). Phylogenetic analyses based on 16S rDNA showed that *Cardinium* strains detected in *Culicoides* were almost identical, as was reported in previous studies [17–19]. The isolates detected in Spain consolidate the presence of the new *Cardinium* C-group that apparently is specific of *Culicoides* biting midges at a global scale.

Endosymbiont based research, especially involving *Wolbachia*, has proven to be a promising technique to control some arthropod borne diseases. One of the most successful research lines involves the use of the life-shortening *w*MelPop *Wolbachia* strain. This strain has shown an important reduction of the lifespan of certain arthropods [34]. Moreover, the *w*Mel *Wolbachia* strain has proven to induce refractoriness towards certain pathogens in mosquitoes [35, 36]. Thus, *Wolbachia* strains of *Culicoides* need to be functionally screened for properties targeting the reduction of either vector’s fitness or pathogen transmission. *Wolbachia* horizontal transmission between different host species has been proposed because of the phylogenetic incongruence between hosts and *Wolbachia* strains [37]. This was proven to be feasible by inducing stable infections in naïve arthropod populations [38]. Therefore, new findings could have an important impact beyond *Culicoides* arena because of the successful transfection of *Wolbachia* even across genus and species barriers.

## Conclusions

The presence of natural infections of *Wolbachia* and *Cardinium* endosymbionts in *Culicoides* deserves attention. The finding might represent the starting point to address new research for the control of *Culicoides*-transmitted diseases. *Wolbachia* can control vector fitness and vector’s pathogen transmission, thus being a potential target for population reduction and replacement strategies. The results of the present study could have an impact beyond *Culicoides* arena because successful *Wolbachia* transfection is possible across genus and species barriers.

## Additional files

Additional file 1: Table S1. NCBI published sequences used to better reconstruct the evolutionary relationship of *Wolbachia* and *Cardinium* isolates. (DOCX 48 kb)

Additional file 2: Table S2. Diagnostic tests performed in *Culicoides* to detect *Wolbachia* and *Cardinium* endosymbionts. Infection frequencies are shown in parentheses for populations where endosymbionts were detected in *Culicoides*. (DOCX 47 kb)

## Abbreviations

BT: Bluetongue
BTV: Bluetongue virus
CDC: Center for Disease Control
PR: Prevalence ratio
SBV: Schmallenberg virus
*wsp*: *Wolbachia* surface protein gene
