# Supplementary material for: First detection of Wolbachia-infected Culicoides (Diptera: Ceratopogonidae) in Europe: Wolbachia and Cardinium infection across Culicoides communities revealed in Spain
Source: Parasit Vectors. 2017 Nov 23;10:582. doi: 10.1186/s13071-017-2486-9 (PMC5701505; doi:10.1186/s13071-017-2486-9)
Supplement: Supplementary file 1 — NCBI published sequences used to better reconstruct the evolutionary relationship of Wolbachia and Cardinium isolates. (DOCX 48 kb) [file 13071_2017_2486_MOESM1_ESM.docx]

**Additional file 1**

**Table S1.** NCBI published sequences used to better reconstruct the evolutionary relationship of *Wolbachia* and *Cardinium* isolates.

| **Accession nº** | **Endosymbiont** | **Gene** | **Host** | **Reference** |
| --- | --- | --- | --- | --- |
| AF020080 | *Wolbachia* | wsp | *Laodelphax striatellus* | Zhou et al., 1998 |
| AF020084 | *Wolbachia* | wsp | *Trichogramma deion* | Zhou et al., 1998 |
| AF020083 | *Wolbachia* | wsp | *Tribolium confusum* | Zhou et al., 1998 |
| AF020085 | *Wolbachia* | wsp | *Tagosedes orizicolus* | Zhou et al., 1998 |
| AF020076 | *Wolbachia* | wsp | *Cadra cautella* | Zhou et al., 1998 |
| KR026947 | *Wolbachia* | wsp | *Culicoides narrabeenensis* | Mee et al., 2015 |
| KR026949 | *Wolbachia* | wsp | *Culicoides narrabeenensis* | Mee et al., 2015 |
| KR026948 | *Wolbachia* | wsp | *Culicoides narrabeenensis* | Mee et al., 2015 |
| KR026950 | *Wolbachia* | wsp | *Culicoides narrabeenensis* | Mee et al., 2015 |
| KR026943 | *Wolbachia* | wsp | *Culicoides brevitarsis* | Mee et al., 2015 |
| AF020061 | *Wolbachia* | wsp | *Culex pipiens* | Zhou et al., 1998 |
| AF020060 | *Wolbachia* | wsp | *Culex quinquefasciatus* | Zhou et al., 1998 |
| AF020059 | *Wolbachia* | wsp | *Aedes albopictus* | Zhou et al., 1998 |
| AF020069 | *Wolbachia* | wsp | *Drosophila simulans* | Zhou et al., 1998 |
| AF020074 | *Wolbachia* | wsp | *Drosophila simulans* | Zhou et al., 1998 |
| KR026951 | *Wolbachia* | wsp | *Culicoides parvimaculatus* | Mee et al., 2015 |
| KR026944 | *Wolbachia* | wsp | *Culicoides brevitarsis* | Mee et al., 2015 |
| KR026945 | *Wolbachia* | wsp | *Culicoides brevitarsis* | Mee et al., 2015 |
| KR026942 | *Wolbachia* | wsp | *Culicoides antennalis* | Mee et al., 2015 |
| AF020067 | *Wolbachia* | wsp | *Drosophila simulans* | Zhou et al., 1998 |
| KR026946 | *Wolbachia* | wsp | *Culicoides bundyensis* | Mee et al., 2015 |
| AF020063 | *Wolbachia* | wsp | *Drosophila melanogaster* | Zhou et al., 1998 |
| AF020066 | *Wolbachia* | wsp | *Drosophila melanogaster* | Zhou et al., 1998 |
| AF020065 | *Wolbachia* | wsp | *Drosophila melanogaster* | Zhou et al., 1998 |
| AF020064 | *Wolbachia* | wsp | *Drosophila melanogaster* | Zhou et al., 1998 |
| AF020072 | *Wolbachia* | wsp | *Drosophila melanogaster* | Zhou et al., 1998 |
| AF020079 | *Wolbachia* | wsp | *Glossina morsitans* | Zhou et al., 1998 |
| AF020081 | *Wolbachia* | wsp | *Nasonia vitripennis* | Zhou et al., 1998 |
| AF020078 | *Wolbachia* | wsp | *Glossina morsitans centralis* | Zhou et al., 1998 |
| AF020070 | *Wolbachia* | wsp | *Drosophila simulans* | Zhou et al., 1998 |
| AF020062 | *Wolbachia* | wsp | *Drosophila auraria* | Zhou et al., 1998 |
| AF020071 | *Wolbachia* | wsp | *Muscidifurax uniraptor* | Zhou et al., 1998 |
| AF020077 | *Wolbachia* | wsp | *Glossina austeni* | Zhou et al., 1998 |
| AF020082 | *Wolbachia* | wsp | *Phlebotomus papatasi* | Zhou et al., 1998 |
| AF020075 | *Wolbachia* | wsp | *Cadra cautella* | Zhou et al., 1998 |
| AF020068 | *Wolbachia* | wsp | *Drosophila simulans* | Zhou et al., 1998 |
| AF020073 | *Wolbachia* | wsp | *Drosophila sechellia* | Zhou et al., 1998 |
| AY327472 | *Cardinium* | 16S | *Plagiomerus diaspidis* | Nakamura et al., 2009 |
| AF319783 | *Cardinium* | 16S | *Encarsia pergandiella* | Nakamura et al., 2009 |
| AY331187 | *Cardinium* | 16S | *Encarsia hispida* | Nakamura et al., 2009 |
| AY327469 | *Cardinium* | 16S | *Aspediotus paranerii* | Nakamura et al., 2009 |
| AB241132 | *Cardinium* | 16S | *Tetranychus urticae* | Nakamura et al., 2009 |
| AF350221 | *Cardinium* | 16S | *Brevipalpus phoenicis* | Nakamura et al., 2009 |
| AB001518 | *Cardinium* | 16S | *Ixodes scapularis* | Nakamura et al., 2009 |
| AY753170 | *Cardinium* | 16S | *Metaseiulus occidentalis* | Nakamura et al., 2009 |
| AB241129 | *Cardinium* | 16S | *Eotetranychus suginamensis* | Nakamura et al., 2009 |
| AB241135 | *Cardinium* | 16S | *Tetranychus pueraricola* | Nakamura et al., 2009 |
| AB241131 | *Cardinium* | 16S | *Amphitetranychus quercivorus* | Nakamura et al., 2009 |
| AB241130 | *Cardinium* | 16S | *Oligonychus ilicis* | Nakamura et al., 2009 |
| AB506775 | *Cardinium* | 16S | *Euides speciosa* | Nakamura et al., 2009 |
| AB506773 | *Cardinium* | 16S | *Harmalia sirokata* | Nakamura et al., 2009 |
| AB506774 | *Cardinium* | 16S | *Sogatella furcifera* | Nakamura et al., 2009 |
| AB506777 | *Cardinium* | 16S | *Culicoides lungchiensis* | Nakamura et al., 2009 |
| AB506776 | *Cardinium* | 16S | *Culicoides arakawae* | Nakamura et al., 2009 |
| AB506778 | *Cardinium* | 16S | *Culicoides ohmorii* | Nakamura et al., 2009 |
| AB506780 | *Cardinium* | 16S | *Amoebophilus asiaticus* | Nakamura et al., 2009 |
| AB506779 | *Cardinium* | 16S | *Culicoides peregrinus* | Nakamura et al., 2009 |
| HG531389 | *Cardinium* | 16S | *Culicoides pulicaris* | Lewis et al., 2014 |
| HG380245 | *Cardinium* | 16S | *Culicoides punctatus* | Lewis et al., 2014 |
| KR026923 | *Cardinium* | 16S | *Culicoides williwilli* | Mee et al., 2015 |
| KR026922 | *Cardinium* | 16S | *Culicoides williwilli* | Mee et al., 2015 |
| KR026921 | *Cardinium* | 16S | *Culicoides victoriae* | Mee et al., 2015 |
| KR026920 | *Cardinium* | 16S | *Culicoides victoriae* | Mee et al., 2015 |
| KR026919 | *Cardinium* | 16S | *Culicoides parvimaculatus* | Mee et al., 2015 |
| KR026917 | *Cardinium* | 16S | *Culicoides narrabeenensis* | Mee et al., 2015 |
| KR026918 | *Cardinium* | 16S | *Culicoides parvimaculatus* | Mee et al., 2015 |
| KR026916 | *Cardinium* | 16S | *Culicoides multimaculatus* | Mee et al., 2015 |
| KR026915 | *Cardinium* | 16S | *Culicoides multimaculatus* | Mee et al., 2015 |
| KR026914 | *Cardinium* | 16S | *Culicoides marksi* | Mee et al., 2015 |
| KR026913 | *Cardinium* | 16S | *Culicoides imicola* | Mee et al., 2015 |
| KR026912 | *Cardinium* | 16S | *Culicoides imicola* | Mee et al., 2015 |
| KR026911 | *Cardinium* | 16S | *Culicoides henryi* | Mee et al., 2015 |
| KR026910 | *Cardinium* | 16S | *Culicoides henryi* | Mee et al., 2015 |
| KR026906 | *Cardinium* | 16S | *Culicoides brevitarsis* | Mee et al., 2015 |
| KR026907 | *Cardinium* | 16S | *Culicoides brevitarsis* | Mee et al., 2015 |
| KR026908 | *Cardinium* | 16S | *Culicoides bundyensis* | Mee et al., 2015 |
| KR026909 | *Cardinium* | 16S | *Culicoides bundyensi* | Mee et al., 2015 |
| JN166962 | *Cardinium* | 16S | *Culicoides oxystoma* | Morag et al., 2012 |
| JN166961 | *Cardinium* | 16S | *Culicoides imicola* | Morag et al., 2012 |
