# Supplementary material for: First detection of Wolbachia-infected Culicoides (Diptera: Ceratopogonidae) in Europe: Wolbachia and Cardinium infection across Culicoides communities revealed in Spain
Source: Parasit Vectors. 2017 Nov 23;10:582. doi: 10.1186/s13071-017-2486-9 (PMC5701505; doi:10.1186/s13071-017-2486-9)
Supplement: Supplementary file 2 — Diagnostic tests performed in Culicoides to detect Wolbachia and Cardinium endosymbionts. Infection frequencies are shown in parentheses for populations where endosymbionts were detected in Culicoides. (DOCX 47 kb) [file 13071_2017_2486_MOESM2_ESM.docx]

**Additional file 2**

**Table S2.** Diagnostic tests performed in *Culicoides* to detect *Wolbachia* and *Cardinium* endosymbionts. Populations where endosymbionts were detected in *Culicoides* are shown in red and their infection frequencies are shown in parentheses.

|  | WOLBACHIA | | | | | | | |  | CARDINIUM | | | | | | | |
| --- | --- | --- | --- | --- | --- | --- | --- | --- | --- | --- | --- | --- | --- | --- | --- | --- | --- |
|  | IMI | | OBS | | PUL | | OTHER | |  | IMI | | OBS | | PUL | | OTHER | |
|  | Livestock  premise | Natural  habitat | Livestock  premise | Natural  habitat | Livestock  premise | Natural  habitat | Livestock  premise | Natural  habitat |  | Livestock  premise | Natural  habitat | Livestock  premise | Natural  habitat | Livestock  premise | Natural  habitat | Livestock  premise | Natural  habitat |
| **Andalucía** | **1/49** | **5/100** | **0/50** | **0/80** | **-** | **47/101** | **-** | **0/2** |  | **0/50** | **0/100** | **0/50** | **0/80** | **-** | **0/100** | **-** | **0/2** |
| Almoraima | - | 5/50 (0,1) | - | 0/30 | - | 47/51 (0,92) | - | 0/2 |  | - | 0/50 | - | 0/30 | - | 0/50 | - | 0/2 |
| Juanar | - | 0/50 | - | 0/50 | - | 50 | - | - |  | - | 0/50 | - | 0/50 | - | 0/50 | - |  |
| Santa Clara | 1/49 (0,02) | - | 0/50 | - | - | - | - | - |  | 0/50 | - | 0/50 | - | - | - | - |  |
| **Astúrias** | **-** | **-** | **0/10** | **2/105** | **-** | **2/100** | **-** | **0/3** |  | **-** | **-** | **0/10** | **1/101** | **-** | **0/100** | **-** | **0/2** |
| Colunga | - | - | - | 0/54 | - | 0/50 | - | 0/2 |  | - | - | - | 1/51 (0,02) | - | 0/50 | - | 0/1 |
| Proaza | - | - | 0/10 | 2/51 (0,04) | - | 2/50 (0,04) | - | 0/1 |  | - | - | 0/10 | 0/50 | - | 0/50 | - | 0/1 |
| **Castilla Mancha** | **0/50** | **0/40** | **-** | **0/70** | **-** | **7/51** | **-** | **0/3** |  | **0/50** | **0/40** | **-** | **0/70** | **-** | **0/50** | **-** | **1/2** |
| La Morera | - | - | - | 0/20 | - | - | - | 0/1 |  | - | - | - | 0/20 | - | - | - | 1/1 (1,00) |
| Quintos de Mora | - | 0/40 | - | 0/50 | - | 7/51 (0,14) | - | 0/2 |  | - | 0/40 | - | 0/50 | - | 0/50 | - | 0/1 |
| Pozo Estanco | 0/50 | - | - | - | - | - | - | - |  | 0/50 | - | - | - | - | - | - |  |
| **Catalunya** | **0/18** | **-** | **0/102** | **0/50** | **0/5** | **0/52** | **3/41** | **-** |  | **0/10** | **-** | **1/92** | **0/50** | **0/3** | **0/50** | **8/40** | **-** |
| Alguaire |  |  |  |  |  |  |  |  |  | - | - | - | - | - | - | 0/1 | - |
| Amposta | - | - | - | - | - | - | 0/1 | - |  | - | - | - | - | - | - | 1/1 (1,00) | - |
| Aramunt | 0/2 | - | 0/12 | - | 0/2 | - | 1/9 (0,11) | - |  | 0/2 | - | 0/4 | - | **-** | **-** | 1/9 (0,11) | - |
| Bonastre | 0/2 | - | - | - | - | - | - | - |  | - | - | - | - | - | - | - | - |
| Brunyola | - | - | - | - | 0/2 | - | - | - |  | - | - | - | - | - | - | - | - |
| Caldes de Malavella | 0/8 | - | 0/12 | - | - | - | 0/4 | - |  | 0/7 | - | 0/12 | - | - | - | 2/4 (0,50) | - |
| Canyamars |  | - | - | - | - | - | 0/2 | - |  | - | - | - | - | - | - | 0/2 | - |
| Garcia | 0/1 | - | 0/1 | - | - | - | 0/3 | - |  | - | - | - | - | - | - | 0/3 | - |
| La Galera | 0/1 | - | - | - | - | - | 0/3 | - |  | - | - | - | - | - | - | 1/3 (0,33) | - |
| Massanes | - | - | 0/70 | - | - | - | - | - |  | - | - | 1/70 (0,01) | - | - | - | - | - |
| Piera | 0/2 | - | - | - | - | - | 0/1 | - |  | - | - | - | - | - | - | 0/1 | - |
| Roda de Barà | - | - | - | - | - | - | 0/1 | - |  | - | - | - | - | - | - | 1/1 (1,00) | - |
| Sant Iscle de Vallalta | - | - | 0/5 | - | - | - | - | - |  |  | - | 0/5 | - | - | - | - | - |
| Sant Just Desvern | 0/1 | - | - | - | - | - | - | - |  | 0/1 | - | - | - | - | - | - | - |
| Susqueda | - | - | 0/2 | - | - | 0/52 | 1/9 (0,11) | - |  | - | - | 0/1 | - | 0/2 | - | 1/7 (0,14) | - |
| Terrades | - | - | - | 0/50 | 0/1 | - | - | - |  | - | - | - | 0/50 | - | 0/50 | - | - |
| Vilanova de la Muga | 0/1 | - | - | - | - | - | 1/8 (0,12) | - |  |  |  |  |  | 0/1 | - | 1/8 (0,12) | - |

Definition of species groups: IMI, *C. imicola*; OBS, *C. obsoletus* s.l.; PUL, *C. pulicaris* s.l.; OTHER; other *Culicoides* species
